# A Motion-based Device Urinary Incontinence Treatment: A Longitudinal Analysis at 18 and 24 Months

**DOI:** 10.1007/s00192-023-05721-z

**Published:** 2024-01-22

**Authors:** Milena M. Weinstein, Gena C. Dunivan, Noelani M. Guaderrama, Holly E. Richter

**Affiliations:** 1 Department of Obstetrics, Gynecology and Reproductive Biology, Division of Female Pelvic Medicine and Reconstructive Surgery, Massachusetts General Hospital, Harvard Medical School, Boston, MA USA; 2 University of Alabama at Birmingham, Birmingham, AL USA; 3 Southern California Permanente Medical Group, Irvine, CA USA

**Keywords:** Urinary incontinence, Motion-based biofeedback, Conservative treatment

## Abstract

**Introduction and hypothesis:**

There are sparse data regarding the long-term efficacy of pelvic floor muscle training (PFMT) for the treatment of urinary incontinence (UI). The objective of this study was to evaluate the impact of an 8-week PFMT program guided by a motion-based intravaginal device versus a standard home program over 24 months.

**Methods:**

Between October 2020 and March 2021, a total of 363 women with stress or stress-predominant mixed UI were randomized and completed an 8-week PFMT program using a motion-based intravaginal device (intervention group) or a home program following written/video instructions (control group). Participants were not asked to continue training after the 8-week program. At 18 and 24 months’ follow-up, the Urogenital Distress Inventory, short-form (UDI-6) and Patient Global Impression of Improvement (PGI-I) were collected. In the original trial, a total of 139 participants in each arm were needed to detect a 0.3 effect size (alpha = 0.05, power 0.8, one-tailed *t* test) in the difference in UDI-6 scores.

**Results:**

A total of 231 participants returned 24-month data. Mean age at 24 months was 51.7 ± 14.5 years, and mean BMI was 31.8 ± 7.4 kg/m^2^. Mean change in UDI-6 scores from baseline to 24 months was greater in the intervention group than the control group (−21.1 ± 24.5 vs −14.8 ± 19.4, *p* = 0.04). Reported improvement using PGI-I was greater in the intervention group than in the control group at 24 months (35% vs 22%, *p* = 0.03, OR 1.95(95% CI 1.08, 3.57).

**Conclusions:**

Pelvic floor muscle training guided by a motion-based prescription intravaginal device yielded durable and significantly greater UI symptom improvement than a standard home program, even in the absence of continued therapy.

**Supplementary information:**

The online version contains supplementary material available at 10.1007/s00192-023-05721-z

## Introduction

Urinary incontinence (UI) is a prevalent health condition, with over 78 million women in the USA reporting bothersome symptoms [1]. UI has negative impacts on physical and psychosocial health and conveys significant economic burden to individuals and society [2–6]. First-line care for all major subtypes of UI (stress, urgency, and mixed) includes pelvic floor muscle training (PFMT) [7], and yet only a small percentage of women undergo PFMT prior to advancing to medications, procedures, or surgical interventions to address their symptoms [8]. Many women do not engage in first-line care at all, instead receiving more advanced treatments such as medication or surgery as their first treatment [8]. There are many explanations for this failure to utilize first-line care, including a lack of confidence in PFMT that has been expressed by clinicians, skepticism regarding efficacy by patients themselves, and difficulty accessing care owing to constraints of time, cost, or provider availability [9–11]. Advances in technology are helping to define new pathways for the implementation of PFMT for UI, making access to effective PFMT available to more women [12–14]. In a previous randomized controlled trial, the superiority of a prescription motion-based device over home PFMT with written and audio instructions for the treatment of stress and mixed UI was demonstrated following an 8-week program [15]. These results were found to be durable over a 12-month period without continued PFMT [15].

The objective of this longitudinal follow-up study was to evaluate the continued efficacy of an initial 8-week regimen of PFMT 24 months after randomization, comparing the use of a prescription motion-based device with a standard home PFMT program in women with stress UI or stress-dominant mixed UI.

## Materials and Methods

This is an 18- and 24-month planned follow-up of a prospective, randomized controlled trial to evaluate the safety and efficacy of a motion-based device (intervention group) compared with a program of standardized home PFMT (control group) in the treatment of stress UI and stress-dominant mixed UI. The trial was registered with http://clinicaltrials.gov, registration: NCT04508153. The study was approved by the Western Institutional Review Board (Study No.1287912).

The study protocol, the initial 8-week study, and 6- and 12-month follow-up data have been reported previously [15–17]. In the original study, enrolled between October 2020 and March 2021, participants with stress UI or stress-dominant mixed UI were recruited via social media for enrollment in a study executed remotely. Participants were randomized using a 1:1 block randomization scheme to perform PFMT using a prescription motion-based device (Leva Pelvic Health System, Axena Health, Auburndale, MA, USA) or according to standardized written and audiovisual instructions adapted from the Voices of PFD website [18].

All participants were mailed information or the device based on their assignment, received three scheduled, scripted phone calls, and performed PFMT according to their study assignment for 8 weeks. They were not specifically asked to continue PFMT following this time period. Primary outcomes in the original study compared the difference between change in the Urogenital Distress Inventory, Short Form (UDI-6) scores in the intervention and control groups from baseline to 8 weeks and change in the number of urgency and stress UI episodes reported in a 3-day diary from baseline to 8 weeks. The 6- and 12-month longitudinal follow-up compared changes in mean scores on multiple validated questionnaires, including UDI-6 and Patient Global Impression of Improvement (PGI-I), from baseline and from 8 weeks for the ongoing evaluation of UI symptoms.

For the planned 18- and 24-month follow-up, surveys were collected, including the UDI-6, a validated measure of bother and severity of UI symptoms [19], PGI-I, Patient Global Impression of Severity (PGI-S), the Pelvic Floor Impact Questionnaire (PFIQ), which includes the Incontinence Impact Questionnaire, Short Form (IIQ-7) subscale; additional subscales of the Pelvic Floor Distress Inventory Short Form (PFDI-20) including the Pelvic Organ Prolapse Distress Inventory-6 (POPDI-6); the Colorectal Anal Distress Inventory-8 (CRADI-8); and the Pelvic Organ Prolapse/Urinary Incontinence Sexual Questionnaire, International Urogynecological Association-Revised (PISQ-IR). Participants were not specifically asked to continue PFMT after the initial 8-week study, i.e., no maintenance regimen was required as part of the study. Performance of PFPT was not monitored in either arm; however, any use of the intervention device was captured by the passive collection of adherence data within the device. Upon completion of each follow-up period, participants were compensated with $100.

Demographics were reported at 18 and 24 months, including reporting of race and ethnicity to ensure the participant population was representative of women with UI, and to evaluate for potential changes in the composition of the group who continued to provide data at 18 and 24 months. Survey results (UDI-6, PFIQ, PFDI-20, PISQ-IR) were evaluated by calculating mean scores. UDI-6 scores were evaluated against the Patient Acceptable Symptom State (PASS) for the survey [20] and converted to UDI Long Form scores to determine if score changes in each group met the minimal clinically important difference (MCID) of 11 points [21, 22]. Overall improvement for the PGI-I was defined as “very much better” or “much better.” Paired *t* tests were used to determine differences between timepoints for within-group differences, and Student’s *t* tests were used to evaluate the differences between groups, as indicated for continuous variables. For categorical variables (PGI-I and PGI-S), Chi-squared tests were used. Statistical analyses were computed using R 1.4.1103.

## Results

**Fig. 1:**
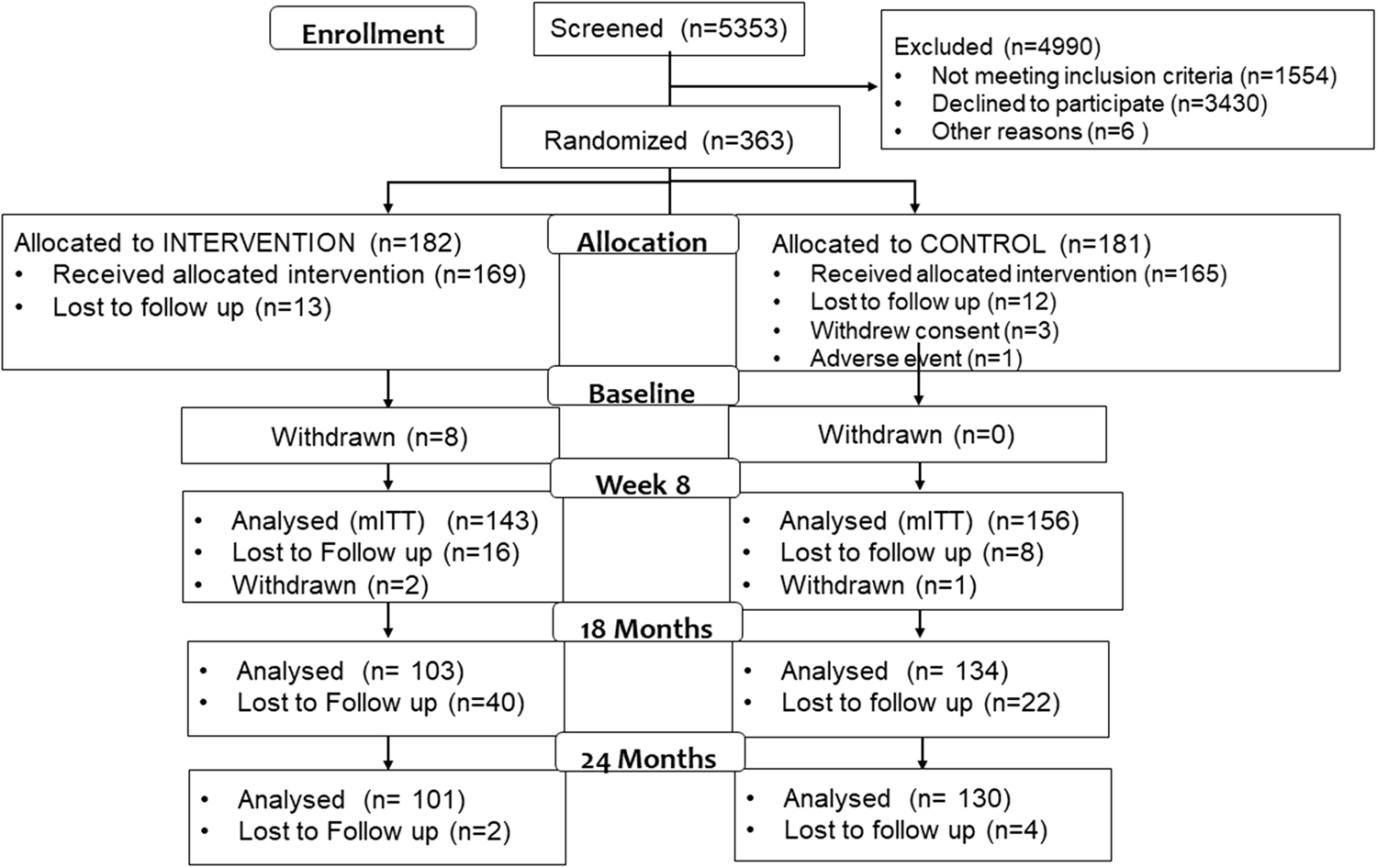
Consolidated standards of reporting trials diagram

At 18 months, 79% of those who were analyzed at 8 weeks (237 out of 299, the modified intention to treat (mITT) population) provided data, and at 24 months, 77% (231 out of 299) provided data. The Consolidated Standards of Reporting Trials diagram is provided in Fig. 1. There were no statistically significant differences in clinical and demographic data between groups at 18 (Appendix 1) or 24 months (Table 1), and there were no significant differences between those who did and those who did not provide follow-up information at 18 or 24 months, including differences in age, race, body mass index (BMI, weight in kilograms divided by height in meters, squared), prior pregnancies, menopausal status, or baseline UDI-6 scores (data not shown). At 24 months the average age of respondents was 51.7 ± 14.5 years, and mean BMI was 31.8 ± 7.4 kg/m^2^. Most participants were postmenopausal and parous.

**Table 1:** Subject demographics and clinical characteristics at 24 months

Demographic	Statistics	Control, *n* = 130	Intervention, *n* = 101	Total, *n* = 232	*p* value
Age	Mean ± SD	51.4 ± 12.1	52.0 ± 12.9	51.7 ± 14.5	0.74
Race, *n* (%)	Asian	5 (3.9)	0 (0)	5 (2.2)	0.12
	Black	12 (9.2)	13 (12.9)	25 (10.8)	
	Whilte	108 (83.1)	78 (77.2)	186 (80.5)	
	Middle Eastern/North African	0 (0.0)	1 (1.0)	1 (0.4)	
	Multi	2 (1.5)	4 (4.0)	6 (2.6)	
	Other^b^	3 (2.3)	4 (4.0)	7 (3.0)	
	Unknown^b^	0 (0.0)	1 (1.0)	1 (0.4)	
Ethnicity, *n* (%)	Hispanic/Latino	10 (7.7)	10 (9.9)	20 (8.7)	0.48
	Not Hispanic/Latino	120 (92.3)	90 (89.1)	210 (60.9)	
	Declined to answer	0 (0.0)	1 (1.0)	1 (0.4)	
BMI (kg/m^2^)	Mean ± SD	32.0 ± 7.6	31.5 ± 7.1	31.8 ± 7.4	0.62
Parity	Median (IQR)	2 (1–4)	3 (2–3)	2 (1–4)	0.36
Mode of delivery, *n* (%)	Vaginal	59 (45.4)	56 (55.5)	115 (49.9)	0.78
	Forceps/vacuum	30 (23.1)	23 (22.8)	53 (22.9)	
	Cesarean section	18 (13.9)	10 (9.9)	28 (12.1)	
Menopausal status^a^, *n* (%)	Post-menopausal	71 (54.6)	57 (56.4)	128 (55.4)	0.15
	Pre-menopausal	59 (45.4)	44 (43.6)	103 (44.6)	

Data are mean ± standard deviation (*SD*), median (interquartile range, *IQR*), or *n* (%) unless otherwise specified

*IQR* interquartile range, *BMI* body mass index

^a^If menopausal status was not specified, participants aged 55 years or older were assumed to be menopausal

^b^Other and Unknown were prespecified categories that participants could choose

**Fig. 2:**
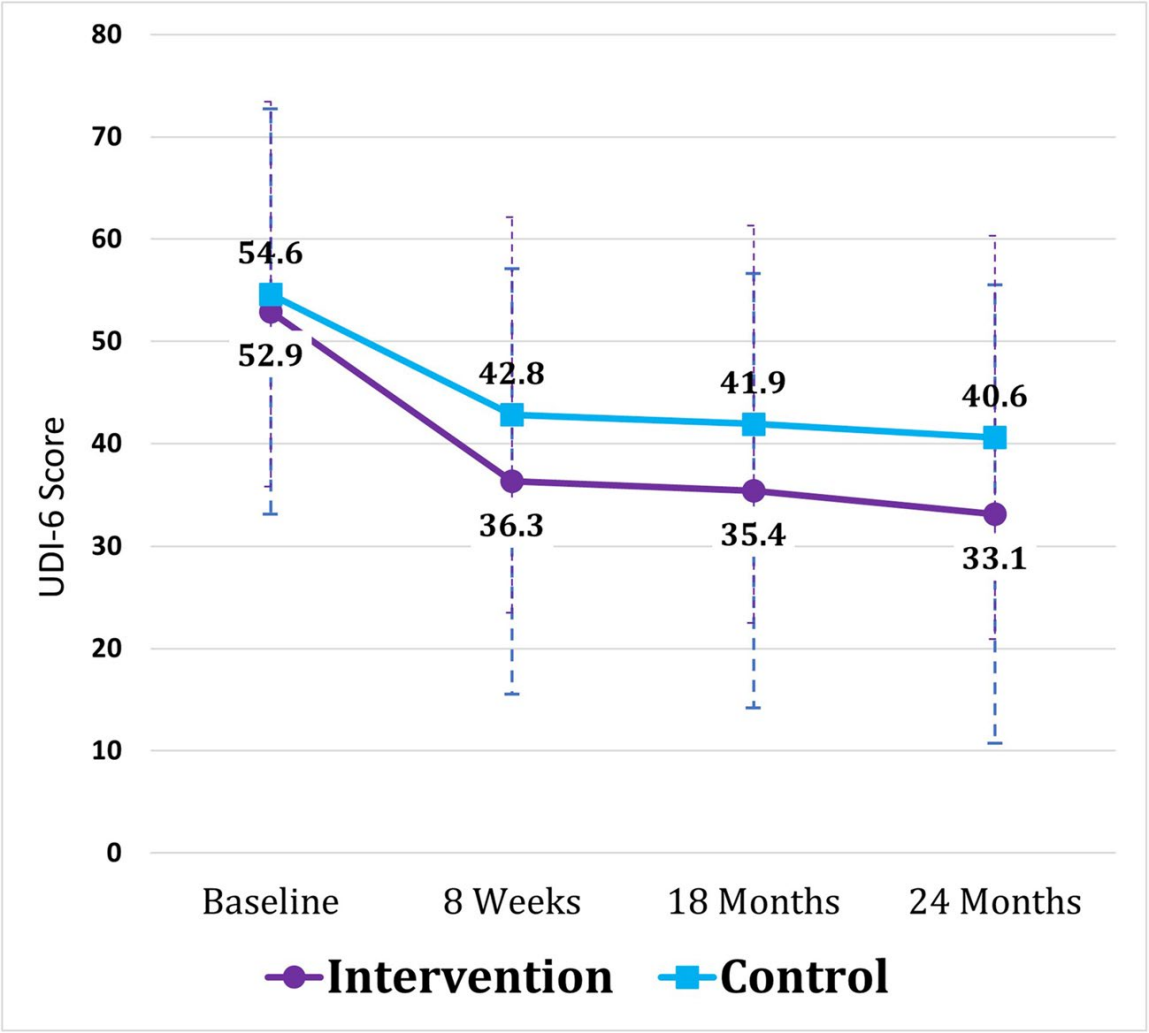
Primary outcome: Urogenital Distress Inventory, short form (UDI-6) score change from baseline to 24 months

Mean UDI-6 score changes were significantly greater in the intervention arm vs the control arm at both 18 (*p* = 0.04) and 24 months (*p* = 0.04) (Table 2). Both groups exceeded the MCID at 18 and 24 months vs baseline, and the difference between groups was equal to or greater than the MCID at both time points. The UDI-6 scores from 8 weeks to 24 months remained stable without deterioration in both groups. The intervention group demonstrated statistically significant improvement from 8 weeks to 24 months (Table 3). This did not meet the MCID, and there was no significant difference between groups over 8 weeks to 24 months. The mean UDI-6 score for the intervention group at 24 months (33.1 ± 22.4) but not the control group (40.6 ± 19.7) met the PASS cutoff of 37.5. The progression of these scores can be seen in Fig. 2.

**Table 2:** Primary outcomes: UDI-6 score change from baseline at 8 weeks, 18 months, and 24 months

	*n*	UDI-6 score change	Within-group *p* value*	Between-group *p* value**	Mean difference between groups, [95% CI]
Baseline to 8 weeks					
Control	156	−14.7 ± 12.2^a^	0.001	0.01	−4.1, [1.0,7.2]
Intervention	143	−18.8 ± 15.0^a^	0.001		
Baseline to 18 months					
Control	134	−13.3 ± 18.5^a^	< 0.001	0.04	−5.6, [−11.0,−0.34]
Intervention	103	−19.0 ± 22.0^a^	< 0.001		
Baseline to 24 months					
Control	130	−14.8 ± 19.4^a^	< 0.001	0.04	−6.3^a^ [−12.1, −0.52]
Intervention	101	−21.1 ± 24.5^a^	< 0.001		

Data are mean ± SD, unless otherwise specified

*UDI-6* Urogenital Distress Inventory, Short Form

*Paired *t* test

**Student's *t* test

^a^Met the minimal clinically important difference

**Table 3:** Interval UDI-6 score change from 8 weeks

	*n*	UDI-6 score change	Within-group *p* value*	Between-group *p* value**	Mean difference between groups, [95% CI]
8 weeks to 18 months					
Control	134	−1.3 ± 16.6	0.39	0.63	−1.2, [−6.1,3.6]
Intervention	103	−2.5 ± 20.5	0.22		
8 weeks to 24 months					
Control	130	−2.5 ± 17.5	0.11	0.43	−2.2, [−7.7, 3.3]
Intervention	101	−4.7 ± 22.6	0.04		

Data are mean ± SD, unless otherwise specified

*UDI-6* Urogenital Distress Inventory, Short Form

*Paired *t* test

**Student's *t* test

The proportion of respondents identifying their symptoms as “much better” or “very much better” on the PGI-I was significantly greater in the intervention group at 18 months (21% vs 36%, *p* = 0.01, OR 2.15, [95% CI 1.19, 3.91]) and 24 months (22% vs 35%, *p* = 0.03, OR 1.95 [95% CI 1.08, 3.57]).

Quality of life and additional pelvic floor disorder symptom scales demonstrated persistent significant improvement from baseline in both groups (Table 4), and there was no significant difference between groups. For the PGI-S, 39.3% (48 out of 156) in the control group and 24.5% (24 out of 143) in the intervention group reported moderate or severe disease at 24 months (*p* = 0.46, OR 1.19, [95% CI 0.075, 1.87]).

**Table 4:** Secondary outcome measures at baseline and 24 months

Outcome measure	Baseline, mean ± SD	24 months, mean ± SD	Paired *t* test, *p* value	Students *t* test, *p* value
IIQ-7				
Control	40.6 ± 25.8	23.1 ± 21.8	< 0.001	0.62
Intervention	38.43 ± 25.73	22.0 ± 23.9	< 0.001	
PFIQ				
Control	59.1 ± 51.4	36.7 ± 46.2	< 0.001	0.51
Intervention	58.2 ± 51.8	35.5 ± 48.9	< 0.001	
POPDI-6				
Control	15.2 ± 16.9	11.9 ± 16.3	0.01	0.07
Intervention	15.4 ± 17.6	9.4 ± 17.1	< 0.001	
CRADI-8				
Control	22.1 ± 20.1	14.3 ± 16.5	< 0.001	0.24
Intervention	19.8 ± 20.1	15.5 ± 19.7	0.004	
PISQ-IR				
Control	2.7 ± 0.3	4.1 ± 1.0	< 0.001	0.24
Intervention	2.8 ± 0.3	4.0 ± 1.0	< 0.001	

*SD* standard deviation, *IIQ-7* Incontinence Impact Questionnaire, *PFIQ* Pelvic Floor Impact Questionnaire, *POPDI-6* Pelvic Organ Prolapse Distress Inventory, *CRADI-8* Colorectal Anal Distress Inventory, *PISQ-IR* Pelvic Organ Prolapse/Urinary Incontinence Sexual Questionnaire, International Urogynecological Association-Revised

Adherence in the intervention group was reported automatically with use of the device. Of 143 participants who completed the initial 8-week study period, 12% (17 out of 143) used the device at least once during the period between the 12- and 24-month follow-up. The mean usage over this time was 0.36% ± 1.89, corresponding to four exercise sessions over 1 year. There was no difference in baseline UDI-6 scores (*p* = 0.60) or UDI-6 score improvement from month 12 to 24 (−20.6 (24.9) vs −27.4 (27.7), *p* = 0.60) between those who continued to use the device and those who did not.

Overall, 24 months after the initial trial, 8 participants proceeded with surgical intervention for UI: 7 participants in the control group and 1 in the intervention group. Eight in the control group and 3 in the intervention group reported using medication for UI. No participants in the control group and 1 in the intervention group reported weight loss or laser treatment.

## Discussion

The significantly greater UI symptom improvement achieved by an 8-week program of PFMT using an at home prescription motion-based device compared with a standard home PFMT program was sustained at 18 and 24 months, even in the absence of an ongoing prescribed regimen. At 24 months, the difference in the UDI-6 from baseline exceeded the MCID in both groups, but the difference between the intervention and control groups met the MCID, suggesting clinically superior durability in the intervention group. Participants in the intervention group were nearly twice as likely to report persistent symptom improvement on the PGI-I. UDI-6 scores for the intervention group, but not the control group, remained below the cut-off established for the PASS that has been previously established for the UDI-6.

Although nonsurgical modalities have been employed to treat UI for decades, the durability of nonsurgical approaches for the treatment of UI has not been well studied. There are several randomized controlled trials confirming persistent efficacy of PFMT at 1 year after training when compared with placebo [23, 24], but 2-year follow-up data from randomized controlled trials are limited [25], both in terms of the number of studies providing data after 2 years, and also because of the limited number of study participants in most long-term follow-up data [23]. The current study, with a high degree of participation at 2 years (77%), contributes to understanding of the value of nonsurgical approaches to UI and may be useful in economic and quality-of-life evaluations comparing surgical with nonsurgical approaches over time.

Participants in either arm were not asked to maintain any particular PFMT regimen although they may have performed PFMT on their own. Notably, only 12% of study participants assigned to the intervention arm (17 out of 143) used the device at all during the 2nd year after the active 8-week study period. In a study by Beyar and Groutz [26], the authors report that long-term performance of PFMT after an initial training program was not associated with better outcomes. This aligns with the current study findings that results were persistent without a prescribed maintenance program, and that even among those in the intervention group who used the device during the 2nd year, results did not differ from those who did not use the device. The durability of results after an initial PFMT regimen and in the absence of any prescribed maintenance program are supported broadly by the literature on exercise science and neuromuscular re-education [27, 28]. A study of resistance training outcomes among adults demonstrates persistent gains in muscle strength and power following an initial 12-week resistance training program with a minimal dose of exercise to maintain initial results [29].

In addition to significant UI symptom improvement that was durable in both groups, those in the intervention group maintained superior improvement. It is plausible that the PFMT regimen embedded in the prescription device provided adequate training intensity to yield greater, durable changes in PFM strength and function compared with the training program in the control group. The intervention group completed all training in a standing (i.e., gravity-dependent) position, executing maximum voluntary contractions for up to 15 s for a cycle of five repetitions. The control group, using a standard program, initiated PFMT in a supine (i.e., gravity-minimized) position, completed sub-maximal voluntary contractions for shorter durations. These exercises were self-progressed to increasingly gravity-dependent positions and to greater effort and duration. These differences in exercise intensity may yield greater gains in muscle strength and power in the intervention group and explain some of the differences in results between groups. Additionally, participants in the intervention group received motion-based visual feedback provided by the device, guiding exercise performance. Literature on adherence and health behavior change suggests that this type of feedback might enhance self-efficacy and contribute to greater adherence to exercise programs [13, 30]. Taken together, use of the motion-based device to direct exercise may contribute to long-term efficacy in multiple ways.

Strengths of this study include the large proportion of participants who provided 18- and 24-month data, resulting in robust long-term follow-up in this large, adequately powered RCT. As has been previously published, limitations include a lack of bladder diaries at these timepoints, and the limitations inherent to a remotely conducted study including the absence of a physical examination [17]. Although a more robust, in-person follow-up containing these additional data points may be ideal, ease of access to remotely obtained surveys may have resulted in a larger percentage of participants who engaged in follow-up. The balance between the ease of access for research participants using remote or app-based data collection tools, and the value of in-person evaluation is an ongoing discussion among researchers.

Use of a motion-based at-home device to guide PFMT resulted in superior UI symptom improvement after 2 years when compared with a home PFMT program alone. The durability of the result persisted regardless of further device use during the follow-up period. Women who wish to optimize improvement and durability resulting from nonsurgical therapy for UI, and the clinicians who counsel them, should consider therapy with a motion-based device.

### Supplementary information

Below is the link to the electronic supplementary material.

Supplementary file1 (DOCX 18 KB)

## Data Availability

Deidntified data from the trial (core variables and outcomes) can be made available to investigators who provide a written request to the corresponding author, regarding systematic review and meta-analysis. Decisions regarding data sharing will be made in conjunction with the sponsor. Data will be available for 5 years from the manuscript submission.
