# Supplementary material for: A Motion-based Device Urinary Incontinence Treatment: A Longitudinal Analysis at 18 and 24 Months
Source: Int Urogynecol J. 2024 Jan 22;35(4):803–10. doi: 10.1007/s00192-023-05721-z (PMC11052829; doi:10.1007/s00192-023-05721-z)
Supplement: Supplementary file 1 [file 192_2023_5721_MOESM1_ESM.docx]

Appendix 1. Subject Demographics and Clinical Characteristics at 18 Months

Demographic	Statistics	Control n=134	Intervention n=103	Total n=237	p-value
Age	Mean ±SD	51.5±12.3	52.5±12.7	51.9±12.4	
Race, n (%)	Asian	5(3.7)	0(0)	5(2.1)	0.09
	Black	12(9.0)	12(11.7)	24(10.1)	
	Whilte	112(83.6)	80(77.7)	192(80.0)	
	Middle Eastern/North African	0(0)	1(1.0)	1(0.4)	
	Multi	2(1.5)	5(4.9)	7(3.0)	
	Other*	3(2.2)	4(3.9)	7(3.0)	
	Unknown*	0(0)	1(1.0)	1(0.4)	
Ethnicity, n (%)	Hispanic/Latino	13(9.7)	10(9.7)	23(9.7)	0.72
	Not Hispanic/Latino	121(90.3)	92(89.3)	213(89.9)	
	Declined to answer	0(0)	1(1.0)	1(0.4)	
BMI (kg/m^2)	Mean ±SD	32.0±7.6	31.5±7.0	31.8±7.4	0.63
Parity	Median (IQR)	2 (1,4)	3(2,3)	2(1,4)	0.85
Mode of delivery, n (%)	Vaginal	62 (46.3)	57(55.3)	119(50.2)	0.56
	Forceps/Vacuum	31(23.1)	23(22.3)	54(22.8)	
	Cesarean Section	18(13.4)	11(10.7)	29(23.2)	
Menopausal status† n (%)	Post-menopausal	73(54.5)	60(58.3)	133(56.1)	0.11
	Pre-menopausal	61(45.5)	43(41.8)	104(43.9)	

Abbreviations: IQR = Interquartile range; BMI = Body Mass Index

Data are Mean± Standard Deviation(SD), Median (interquartile range, IQR), or n(%) unless otherwise s[illegible]

*Other and Unknown were prespecified categories that participants could choose

† If menopausal status was not specified, participants aged 55 years or older were assumed to be me[illegible]
